# Bayesian adaptive clinical trials of combination treatments

**DOI:** 10.1016/j.conctc.2017.11.001

**Published:** 2017-11-03

**Authors:** Kristian Thorlund, Shirin Golchi, Edward Mills

**Affiliations:** a Department of Health Research Methods, Evidence, and Impact, McMaster University, Hamilton, ON, Canada; b Department of Statistics and Actuarial Science, Simon Fraser University, Vancouver, BC, Canada

**Keywords:** Adaptive trials, Additivity, Bayesian, Combination treatment

## Abstract

Randomized clinical trials (RCT) increasingly investigate combination therapies. Strong biological rationale or early clinical evidence commonly suggest that the effect of the combination treatment is importantly greater than the maximum effect of any of the individual treatments. While these relationships are commonly well-accepted, RCTs do not incorporate them into the design or analysis plans. We therefore propose a simple Bayesian framework for incorporating the known relationships that the effectiveness of a combination treatment exceeds that of any individual treatment, but does not necessarily exceed the sum of individual effects. We term the collation of these two relationships ‘fractional additivity’. We performed a binary outcome simulation study of a response adaptive randomized three-arm clinical trial with treatment arms A, B, and A&B that allowed for dropping an inferior treatment arm and terminating the trial early for superiority during any of 4 interim analyses. We compared the Bayesian fractional additivity model to a conventional analysis by measuring the expected proportion of failures, sample size at trial termination, time to termination, and root mean squared error of final estimates. We also compared the fractional additivity model to a ‘full additivity’ model where the effect of A&B was assumed to be the sum of the effect of A and B. In simulation scenarios where important fractional additivity or full additivity existed, the Bayesian fractional additivity model yielded a 3–4% relative reduction in expected number of failures, and a 30%–50% relative reduction in sample size at trial termination compared to a conventional analysis. These results held true even when the Bayesian fractional additivity model employed a biased prior. The full additivity model had slightly higher gains, but too frequently terminated the trial at the first interim look. In scenarios where no or weak fractional additivity exists, the expected sample size and time to termination were similar for the Bayesian fractional additivity model with a moderately optimistic bias about fractional additivity and the conventional model. Lastly, the fractional additivity model generally yielded similar or lower root mean squared error compared to the other models. In conclusion, our proposed Bayesian *fractional additivity* model provides an efficient approach for estimating effects of combination treatments in clinical trials. The approach is not only highly applicable in adaptive clinical trials, but also provides added power in a conventional RCT.

## 1 Background

Several clinical trials investigate combinations of interventions that have already been demonstrated to be individually effective. Historically, the superiority of combination therapies (vs single agent therapies) have been demonstrated medical areas such as in cardiovascular diseases (e.g., the poly-pill) and respiratory diseases [3], [4]. Recently, superiority of combination therapies have been definitely demonstrated in phase III randomized clinical trials (RCT) in areas such as immuno-oncology and type II diabetes (see example Box 1 for detailed description) [1], [2], [5], [6]. In these combination therapy RCTs, the effectiveness of the individual interventions is typically well known, and there are typically substantial biological rationale, early clinical evidence, or evidence from related disease areas to suggest that the combination of interventions will work markedly better than any of the interventions alone [3], [4], [7]. However, RCTs of combination treatments commonly analyse a combination therapy arm as if it is a separate individual intervention. For example, this is generally the case in 2 × 2 factorial trials across all areas of medicine. Thus, no advantage is taken of prior knowledge and assumptions about the combination therapy in the conduct and analysis of the clinical trial.

While true additivity (i.e. the property that the effect of the combination of two intervention equals the sum of the two individual treatment effects) is rare, in many cases it is plausible to assume that treatment combinations investigated in clinical trials will exhibit markedly better effects than each of the individual treatments alone. In other words, it is plausible to assume clinically meaningful *fractionally additive* effect of the combination treatment investigated (also previously referred to as *antagonistic additivity* [3], [7]). Thus, structurally incorporating *fractional additivity* into the statistical analysis of a clinical trial should theoretically suffice to optimize the trial design in terms of mitigating sample size requirements and trial duration. Yet to our knowledge, no clinical trial has previously capitalized on prior knowledge about additivity or fractional additivity.

We therefore propose a Bayesian framework for incorporating *fractional additivity* in the statistical model and analysis of clinical trial of combination treatments. The proposed model expresses the effect of a combination treatment, A&B, as the maximum of A and B, plus a *fractional additivity parameter* times the minimum of A and B. A weakly informative prior distribution is assigned to the *fractional additivity* parameter to reflect plausible ranges of *fractional additivity*, yet does not rule out equipoise nor full additivity (also previously referred to as *synergistic additivity*). Due to the hypothesized efficiency gain we apply the proposed Bayesian *fractional additivity* model within an adaptive clinical trial setting. We test the performance of the proposed model against a conventional approach using simulations.

## 2 Methods

We propose a Bayesian *fractional additivity* modelling framework to optimize estimation of additive effects in clinical trials. Under the conjecture that the proposed model adds considerable efficiency compared to the conventional framework, we conduct a simulation study to assess its performance in an adaptive clinical trial setting. In addition, we illustrate the evolution of posterior probabilities informing trial adaptation in 3 simulated clinical trials.

For simplicity, we only consider a binary outcome clinical trial setting in this paper. However, the proposed model can easily be generalized to other types of outcomes (e.g., continuous or time-to-event data).

### 2.1 The Bayesian fractional additivity model

Under the proposed *fractional additivity* model, we make two seminal assumptions:

1) The effect of A&B is likely larger than the maximum of A and B;
2) The effect of A&B is likely smaller than the sum of the effects of A and B.

Letting θ_A_, and θ_B_ denote the log odds of the treatment responses for A and B, respectively, and letting θ_A&B_ denote the log odds treatment response of A&B, we can express θ_A&B_ as a function of θ_A_, and θ_B_ as follows:

(1) θ_A&B_ = max(θ_A_, θ_B_) + *f*·min(θ_A_, θ_B_),

where *f* is likely a number between 0 and 1 that denotes the fraction of additivity that the combination treatment exhibits (note that ‘θ_A&B_ = θ_A_ + θ_B_’ is what is conventionally referred to as ‘full additivity’).

This model is easily set up in a Bayesian framework that places non-informative priors on the effect sizes (i.e., the log odds) of individual treatment effects, θ_A_ and θ_B,_ and a weakly-informative prior on the fractional additivity parameter *f*. The model is fit using RStan version 2.14.1 (Stan is a probabilistic programming language that implements Hamiltonian Monte Carlo and RStan is an R interface to Stan) [8]. The Stan implementation of the model is provided in the supplementary material.

#### 2.1.1 Prior choice for fractional additivity parameter

The parameter *f* represents the fractional additivity. Under the assumptions for model (1), *f* should lie between 0 and 1, and so a first natural choice would be a beta distribution. However, strictly constraining *f* to the (0,1) interval, by the choice of prior, implicitly violates the assumption of equipoise in RCTs. Thus, hard constraints should be avoided to allow deviations from the fractional additivity assumption. We instead propose to use a normal distribution as a prior for *f.* Expert belief can be used to determine the prior mean, while a variance of 0.16 is supposed to introduce sufficient uncertainty under model (1) to allow the accumulating data to shape the inference, while still being sufficiently informative to stabilize and strengthen estimation (see Table 1 for 95% confidence intervals for group responses under this choice of variance). In practice, *f* is not known, but good biological rationale or early clinical evidence is typically available to inform *f's* distribution. In this paper, we specifically test scenarios where the mean prior distribution for *f* is unbiased (i.e., the truth in the simulation), and where *f* is biased positively or negatively by a 25% (see section 4.2 for further details).

**Table 1:** Overview of simulation scenarios.

Simulation Parameter	Fixed values by scenario
Response probabilities for Tx A and Tx B	1 Pr(response with A) = 35%, Pr(response with B) = 40% 2 Pr(response with A) = 40%, Pr(response with B) = 40%
Fractional additivity	1 *f* = 0.50 2 *f* = 0.75 3 *f* = 1.00
Prognostic factor variability	σ^2^ = 0.16 corresponding to 95%CI of group response of: 1 6.8%–80% when Pr(response with A) = 35% 2 8.4%–83% when Pr(response with A) = 40%

### 2.2 Adaptive design

Due to the anticipated efficiencies of the proposed fractional additivity model as well as the Bayesian nature of the model, we propose applying the model within an adaptive trial design framework. For completeness, however, we confirmed the superior power of the model in a conventional parallel design framework (see Fig. S.1 in supplementary material). We consider a three-arm response adaptive randomized (RAR) clinical trial design that allows for 1) continually adapting the allocation ratios by the updated probabilities of superiority for any treatment; 2) dropping of an inferior treatment arm; and 3) early stopping for superiority. At the beginning of the trial patients are assigned to each of the three intervention arms with equal probabilities (1:1:1). Adaptations can in principle be applied anytime new outcome data becomes available. However, for simplicity and computational feasibility we consider 4 interim analyses at which adaptations can be made. The four interim looks are spaced equally between the first patient enrollment and reaching a fixed parallel design sample size requirement between A&B and the maximum of A and B (e.g., 80% power and 5% type I error to detect a 20% difference). Thus, the first interim analysis occurs when outcome data on 20% of this required sample size has been accrued, the second at 40%, and so forth. Trial adaptations are based on the interim posterior probabilities that A, B, and A&B, respectively are better than the two other interventions. Let *p*_*A best*_, *p*_*B best*_, and *P*_*A&B best*_ denote these three probabilities. At each interim look, the allocation proportions are updated to the ratio between the square roots of these three probabilities (i.e., √*p*_*A best*_: √*p*_*B best*_: √*P*_*A&B best*_). The use of square roots rather than crude probabilities avoids too rapid adaptation and has become common place in adaptive trials [9]. We also allow for dropping an inferior treatment arm if the square root probability of superiority falls below 0.01, as well as early termination of the trial for superiority if the probability of superiority exceeds 0.95 at any interim look. If the termination criterion is not met during interim looks, the trial continues until the total sample size is reached.

Note, the above adaptation and termination rules were established via preliminary simulations monitoring evolution of posterior probabilities at a fixed 1:1:1 allocation (see supplementary material Figs. S2 to S.4).

### 2.3 Data model

Data for a three-arm clinical trial are simulated from given values for probability of response in treatment arm A and in treatment arm B, a fractional additivity value, and added variability to introduce potential for confounding by prognostic imbalance at lower sample sizes. For each patient, a treatment is first allocated randomly according to the given allocation proportion. For patient *i*, the probability of response on treatment $X_{i}$ is given by

(2) $$\text{pi}=\text{Pr}\left(\text{response}|X_{i}\right)\;=\text{expit}\left(\theta_{X}\;+\;\varepsilon_{i}\right)\;\text{where}\;\text{∼}\;\text{N}\left(\text{0,}\sigma^{2}\right)\text{,}$$

where $\theta_{X}$ denotes the log odds for treatment X, $\varepsilon_{i}$ is a random offset to impose the well-known risk of prognostic imbalance, and *σ*^*2*^ is the variance of $\varepsilon_{i}$ determining the degree of potential prognostic imbalance. The incorporation of prognostic imbalance via $\varepsilon_{i}$ is similar to that employed in previous simulation studies of clinical trials [10], [11]. Subsequently, the binary response, *r*_*i*_, follows a Bernoulli distribution

(3) r_i_ ∼ Bernoulli(p_i_).

For the treatment A&B, $\theta_{A\&B}$ is calculated using equation (1) with given values of θ_A_, θ_B_, and *f*.

## 3 Illustration of the fractional additivity model

To illustrate the performance of the fractional additivity model under the investigated adaptive clinical trial design, we select two simulated clinical trials representing possible clinical trial settings. These two select examples are simulated with the methods described above. We monitor how the probabilities of superiority for each treatment evolve over time along with the models' estimates of intervention effects, and how these influence adaptation decisions under the considered adaptive design.

The examples were chosen based on the number of interim looks required for a decision to be reached. The two selected examples represent.

a) an example with low early random error yielding a rapid termination of the trial as early interim data strongly agree with the model;
b) an example with high early random error necessitating some interim looks before the fractional additivity assumption is confirmed by the data.

**Fig. 1:**
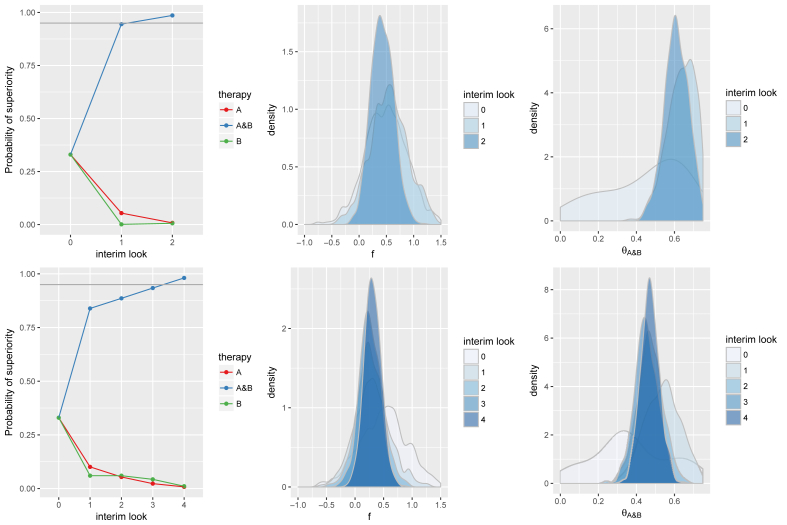
Presents two illustrative examples of the evolution of posterior distributions with the fractionally additive model under the adaptive design: an example where the data shows the expected fractional additive effect early (first row); one example in which fractional additivity is less pronounced due to random error and a larger number of batches is required to identify the superior therapy (second row). The graphs in the first column show the evolution of the probabilities of being the best therapy and the graphs in the second and third columns show the posterior density estimates for the fractional additivity parameter and the effect size of combined treatments evolving as more data are collected.

Fig. 1 presents the evolution of the probabilities of superiority in relation to the evolution of posterior distributions of *f* and θ_A&B_ for each of the above two examples. Fig. S.5 in the supplementary material presents the same for θ_A_ and θ_B_. The speed of adaptation is strongly correlated to the accuracy of estimates of *f*. For example, in scenario a) the posterior distribution of *f* at the first interim look is concentrated around the truth (f=0.5). By contrast, in scenario b) the posterior distribution of *f* converges more slowly and at the fourth interim look where the trial meets the stopping criteria *f* and θ_A&B_ are even slightly downward biased. It is also worth noting the interplay between the evolution of posterior probabilities of *f* and the treatment effects (θ_A_, θ_B_ and θ_A&B_). In scenario a), the first interim look yields an overestimate of θ_A_ and an underestimate of θ_B_, which nonetheless together add up to an overestimate of θ_A&B_. At the second interim look, the biases of θ_A_ and θ_B_ are more negligible. From the first interim overestimate of θ_A&B_ the RAR has ensured a relatively large number of patients enrolled to A&B, thus substantially increasing the precision of A&B, which appears to support a high probability of superiority for A&B thus leading to termination at the second interim look. In scenario b), both θ_B_ and θ_A&B_ start at slight underestimates. In the second interim look θ_A_ is also slightly underestimated, leading to all three parameters being underestimated. While A&B is trending as superior to both A and B in the third interim look, the point estimates do not change, and thus, it is only at the fourth interim look that sufficient precision is available to assert trial termination.

We have selected the above two examples under the case that Pr(response with A) = 40%, Pr(response with B) = 40% and f=0.5. With respect to statistically detecting superiority of A&B, this is a relatively ‘challenging’ scenario as the superiority of A&B to the individual treatments is fairly subtle. Thus, out of all the considered simulations scenarios (see below) the signal to noise ratio is the lowest in this scenario. With larger fractional additivity such as 0.75 or 1 (tested in the simulations) or with Pr(response with A) = 35%, RAR and termination rules are likely more efficient due to the more apparent treatment effect and superiority of A&B.

## 4 Simulation study

### 4.1 Simulation scenarios

To test the empirical performance of the proposed *fractional additivity* model as well as the full additivity model against the conventional approach we run 2000 simulations from the three arm clinical trials data model described above across six select scenarios where meaningful fractional additivity or full additivity existed. These scenarios include pre-set values for probability of response in treatment arms A and B, and given fractional or full additivity values. These are presented in Table 1. In addition, the variance of the prognostic imbalance offset is set to *σ*^*2*^ = *0.16*, which corresponds to reasonably wide distributions for the individual-patient response probabilities (see Table 2). Lastly, across the six chosen combinations of θ_A_, θ_B_, and *f*, we consider the fixed sample size requirement to detect the true difference between A&B and the maximum of A and B with 80% power and 5% type I error (two sided) as last possible point trial termination (under the adaptive design). The ‘true’ difference between A&B and the maximum of A and B is determined by the set simulation scenario values, which are presented in Table 3.

**Table 2:** Overview of analytic approaches tested for each scenario.

Model specification	Description
Statistical model	1 Fractional additivity model: logistic model with θ_A&B_ given by equation (1) 2 Conventional logistic model treating θ_A&B_ as independent of θ_A_ and θ_B_
Priors for *f* (fractional additivity)	1 Normal distribution, mean = scenario *f* (i.e., no bias), variance = 0.16 2 Normal distribution, mean = *f* ± *0.25* (i.e., 25% absolute bias), variance = 0.16
Randomization allocation	1 Adaptive by adjusted posterior probability of being best (see analysis section)) 2 Conventional 1:1:1 ratio (see sensitivity analysis in supplementary material)

**Table 3:** Total fixed sample size requirement for each scenario.

Pr(response with A)	Pr(response with B)	*f*	Total sample size per arm
0.35	0.4	0.50	1745
0.40	0.4	0.50	1637
0.35	0.4	0.75	789
0.40	0.4	0.75	736
0.35	0.4	1	452
0.40	0.4	1	420

In addition to the above simulation scenarios, we also consider cases where no or weak fractional additivity is present, i.e., f=0 and f=0.25. Like the above six scenarios, we consider the fixed sample size requirement to detect the true difference between A&B and the maximum of A and B with 80% power and 5% type I error (two sided) as last possible point trial termination (under the adaptive design). However, since the simulated fractional additivity is either absent or weak, the assumed difference was set to an upward bias of 0.5 added to the true (simulated) fractional additivity (i.e., 0.5, when *f* = *0.25*, and 0.75 when *f* = *0.25*) These four scenarios with no or weak fractional additivity are analysed separately from the first 6 scenarios with meaningful fractional or full additivity mentioned above to represent the situation where the fractional additivity is an incorrect assumption.

### 4.2 Analysis

We compare the Bayesian fractional additivity model (1) with a corresponding conventional Bayesian model in which A&B was considered independent of A and B (i.e., as a third treatment C) as well as a model that incorporates full additivity assumption, i.e. θ_A&B_ = θ_A_ + θ_B_. We also test three sets of priors for the fractional additivity coefficient *f*: a weakly informative prior without bias and a weakly informative prior with 25% optimistic bias and a prior distribution with 25% negative bias (see also Table 2). For the scenarios where no or weak fractional additivity exists, only priors with means 0.5 and 0.75 where used to represent the case that the fractional additivity assumption is incorporated incorrectly via overtly optimistic priors. The models are compared under the described adaptive clinical trial setting in which posterior probabilities are used to drive response adaptive allocation, early termination of arms, and early termination of the trial.

The models are assessed by conventional ethics and efficiency measures in the RAR framework explained above. First, we consider the expected proportion of failures (EPF) and the expected sample size (ESS) at time of termination. In addition to ESS, we also consider the cumulative probabilities of trial termination for all models at each interim look. The rationale for estimating EPF is that when a therapy is less effective than the others, one would hope that the trial is adapted more quickly to assign fewer patients to the inferior arms, thereby decreasing the number of negative outcomes. Note, however, for scenarios with weak or no additivity we do not consider EPF as there is no meaningful ethical gain from allocating more patients to an experimental treatment arm. Second, from the statistical perspective bias and precision of the estimates are common bases of comparison. Therefore, we compute the Root Mean Squared Error (RMSE) for the effect size estimates obtained throughout the trial analysis for the two models. For therapy T the RMSE is given by,

$$RMSE_{T}=\sqrt{\frac{1}{MK}\sum_{m=1}^{M}\sum_{k=1}^{K}{\left({\hat{\theta }}_{\left(mk\right)T}-\theta_{T}\right)}^{2}}\text{,}$$

where M is the number of simulations, K is the number of looks at the data throughout the trial (max of K is 5 if the final look is at the fixed sample size and the trial is not terminated early), $\theta_{T}$ is the true effect size of therapy T and ${\hat{\theta }}_{\left(mk\right)T}$ is the estimated (posterior mean) effect size after interim look k and in simulated trial m.

## 5 Results

**Fig. 2:**
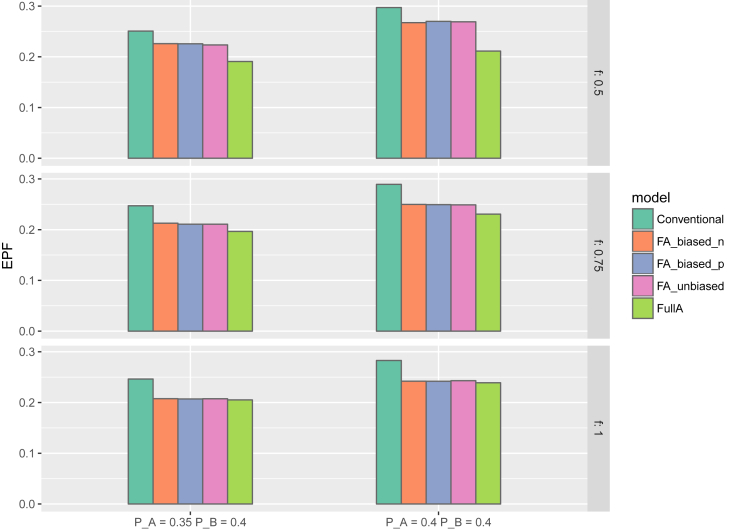
Expected proportion of failures in 6 simulation scenarios for the proposed fractional additivity model with positively and negatively biased and unbiased priors on θ_A&B_, the conventional model (i.e, where θ_A&B_ is treated as completely independent of θ_A_ and θ_B_ and all priors are non-informative) and a model with full additivity assumption (*f* = 1). The upper, middle and lower plots present the scenarios where the *true* fractional additivity parameter value (*f)* is set to 0.5, 0.75, and 1, respectively. The left plots present the scenario where the failure probabilities of treatments A and B are set to *PA* = 0.35 and *P*B = 0.40, whereas the plots to the right present the scenarios where *PA* = 0.40 and *P*B = 0.40.

**Fig. 3:**
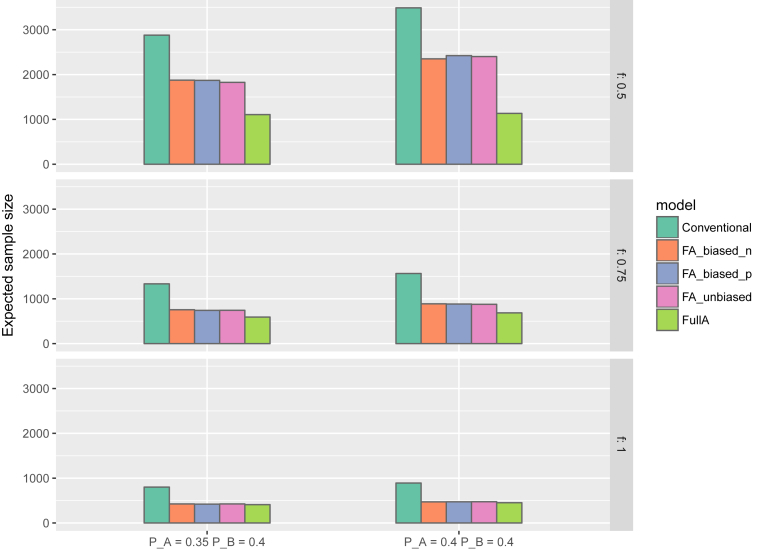
Expected sample size in 6 simulation scenarios for the proposed model with positively and negatively biased and unbiased priors on θ_A&B_, the conventional model (i.e, where θ_A&B_ is treated as completely independent of θ_A_ and θ_B_ and all priors are non-informative) and a model with full additivity assumption (*f* = 1). The upper, middle and lower plots present the scenarios where the *true* fractional additivity parameter value (*f)* is set to 0.5, 0.75, and 1, respectively. The left plots present the scenario where the failure probabilities of treatments A and B are set to *PA* = 0.35 and *P*B = 0.40, whereas the plots to the right present the scenarios where *PA* = 0.40 and *P*B = 0.40.

**Fig. 4:**
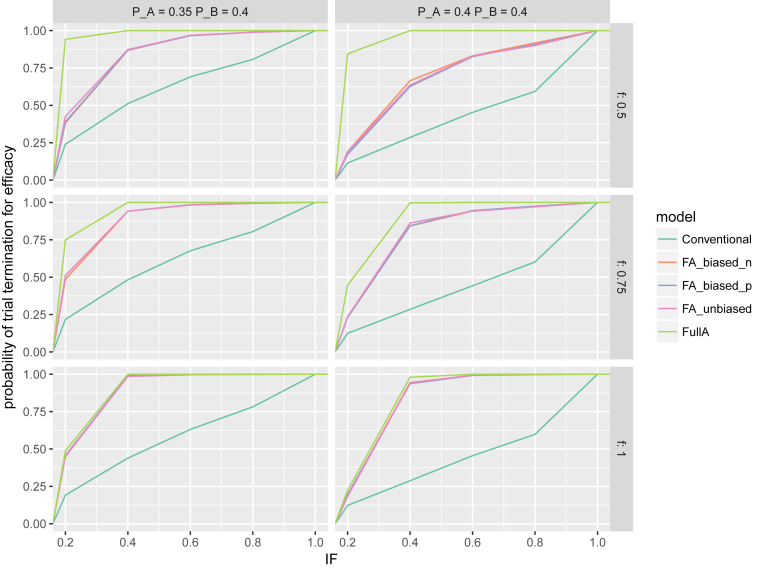
Empirical cumulative distribution function for the probability of trial termination (y-axis) by cumulative Information Fraction (IF) of the required sample size. Note, each interim look accounts for 20% information fraction (e.g., the information fraction of 60% corresponds to the third interim look).

Fig. 2 presents the EFP across the simulation scenarios with meaningful fractional additivity or full additivity (i.e., *f* = *0.5, 0.75,* or *1.0*. Fig. 3, Fig. 4 show the ESS at trial termination and cumulative probabilities of trial termination over interim looks for the scenarios with meaningful or full additivity, and Figs. S.9 to S.10 in the supplementary material present these two plots for scenarios with no or weak fractional additivity. The simulation results for MSE are presented in Figs. S.8 to S.10 (meaningful or full additivity) and Figs. S.11 to S.13 (no or weak fractional additivity) in the supplementary material.

### 5.1 Expected proportion of failures

In all scenarios, the fractional additivity models yielded a lower EPF than the conventional model. Across scenarios this reduction of EPF approximately varied between 3 and 4% of patients. All fractional additivity models (positive prior bias, negative prior bias, no prior bias) were highly similar. The full additivity model attained the smallest EPF, but as stated in Section 5.3, it is due to underestimating the individual treatment effects that the trial is terminated early under this model. The full additivity model yielded an approximate 5% reduction in EPF compared to the conventional model across scenarios.

### 5.2 Expected sample size at trial termination

In the scenarios where meaningful or full additivity was simulated, the ESS at the trial termination was consistently smaller for the fractional additivity models and the full additivity model. Across all scenarios the fractional additivity model approximately reduced the ESS by a relative 30–50%, and the full additivity model by approximately 50–60%. For scenarios where fractional additivity existed (i.e., *f* = *0.5* and 0.75), the full additivity model yielded substantially smaller ESS than the fractional additivity models. For the scenario where full additivity existed (i.e., *f* = 1.00), the ESS were similar across all models. The absolute decrease in ESS varied from approximately 1000 to 4000 patients across the six scenarios. The average time to trial termination was also lower under both the fractional additivity models and the full additivity model. Fig. 4 presents the cumulative probability of trial termination by the cumulative information fraction (i.e., the fraction of required sample size reached). Under all scenarios, the cumulative probability of termination increases more rapidly under the fractional additivity models and full additivity model compared to the conventional model. Similar to the EPF, the ESS values where smaller under the full additivity model due to early termination of the trial resulted from underestimating the individual effects. In the scenario where *f* = 0.5, the full additivity model resulted in trial termination in over 90% of all first interim looks. In scenarios where *f* = 1.0, the fractional additivity models and the full additivity model performed similarly.

In the scenarios where no or weak fractional additivity was simulated, the ESS at termination was approximately 3-fold lower under the full additivity model and the fractional additivity model assuming a 0.75 positive bias. However, ESS of the fractional additivity model assuming a 0.5 positive bias did not differ markedly from the ESS of the conventional model.

### 5.3 Root mean squared error

In the scenarios where meaningful or full additivity was simulated, the RSME estimates of A&B were similar across all models with no difference between any of the models exceeding 0.02. For estimates of A and B, the highest RSME was generally associated with the full additivity model in scenarios of 0.5 fractional additivity, the conventional model was associated with the highest RSME in the scenarios of full additivity. The conventional model generally had slightly higher RSME than the full additivity model in scenarios with fractional additivity of 0.75, although some variation to this trend could be observed. Both had higher RSME than all three fractional additivity models for A and B across all scenarios and were similar for full additivity. Note, however, the sample sizes are larger for the conventional models and RMSEs are not standardized by differences in sample sizes between simulations. As for the full additivity model the RMSEs for the individual effect estimates are systematically larger except for, f = 1, in which case full additivity is the correct assumption. We emphasize the increase in RMSE under the full additivity model is mainly due to the bias in estimating the effect sizes. The fractional additivity model does just as well when full additivity is a correct assumption and therefore it is the recommended model since in practice the full additivity assumption is not verifiable.

## 6 Discussion and conclusion

In this paper, we have proposed a Bayesian *fractional additivity* model, which more efficiently estimates an established superiority of a combination treatment compared with the single agents of the treatment combination. We have demonstrated, via simulation, that the proposed model is highly applicable and efficient under adaptive clinical trial designs. In particular, the proposed model increases power, reduces mean squared error results and results in notable reduction in expected sample size and expected proportion of failures. The model also performs as well as a full additivity when full additivity exists, whereas a full additivity model performs poorly in scenarios where full additivity is not met. Lastly, we have demonstrated via simulation that the proposed fractional additivity model has acceptable statistical properties when no or weak fractional additivity exists. While the proposed model is only presented for binary outcomes, both the model and the simulation results are easily generalizable to other types of outcomes.

To our knowledge, our proposed model is the first model to allow for efficient modelling of *fractional additivity*. The superior performance of the Bayesian *fractional additivity* model over conventional model, both within an adaptive design framework and a conventional RCT framework, was expected as the model is distinctly designed to capitalize on the link between the individual agents and the combination of these. Further, the added information via prior on the fractional additivity parameter *f* is expected to decrease sample size requirements. It is also comforting that under a substantially biased prior (both optimistic and pessimistic) for *f* the model still performs similarly to that of an unbiased prior. Likewise, the superior performance of the *fractional additivity* model over the *full additivity* model was also expected as the underlying assumption of the latter is, of course, violated when full additivity does not exist. The performance of the fractional additivity model (with optimistic priors) under scenarios where no or weak fractional additivity exist suggest a reasonable trade-off between a relatively small risk of stopping early for superiority versus the substantial improvement in efficiency one stands to gain if fractional additivity holds. Of course, it should be recognized that since this paper is the first to propose a Bayesian fractional additivity model, only a limited number of scenarios have been covered. Scenarios with lower population event rates or multi-center clinical trials where large between-center variation exists could prove challenging for the fractional additivity model to adapt to. While we hope to pursue this further is later methods papers, clinical trial investigators employing the proposed model should of course make sure to run a comprehensive set of simulations during the planning of their adaptive trial. Even with this precaution, there are still many examples where pre-trial simulations have concluded one thing, but the opposite finding was observed upon trial termination. Thus, as with any novel method for adaptive clinical trials we can only recommend caution and diligence.

Since the number of clinical trials investigating combination therapies are increasing, our proposed model will have incremental relevance and utility over the next years. Of course, we would recommend scrutiny around applying our model to the specific context. In our simulations, we have applied somewhat arbitrary but general weakly informative priors to the fractional additivity parameter. In practice, prior knowledge (or lack thereof) about the likely degree of fractional additivity may call for use of different priors than the ones applied in this paper. Further, as several clinical trials of combinations will surely also be evaluating efficacy and safety with time-to-event outcomes or continuous outcomes, the proposed model framework should be expanded accordingly. In this vein, equation (1) is easily generalizable as long as a suitable link function can be chosen, but the choice of priors may require further testing in preliminary simulation studies.

In conclusion, the proposed Bayesian *fractional additivity* model provides an efficient approach for estimating effects of combination treatments in clinical trials. The approach is highly applicable in adaptive clinical trials, but also provide added power in conventional settings.

## Appendix A Supplementary data

mmc1 mmc1
