# Supplementary material for: Bayesian adaptive clinical trials of combination treatments
Source: Contemp Clin Trials Commun. 2017 Nov 3;8:227–33. doi: 10.1016/j.conctc.2017.11.001 (PMC5898557; doi:10.1016/j.conctc.2017.11.001)
Supplement: mmc1 [file mmc1.pdf]

Figure S.1: Power of detecting superiority of A&B (i.e., Pr($P_{A\&B\ best}$ >0.95)) for fixed sample size design.

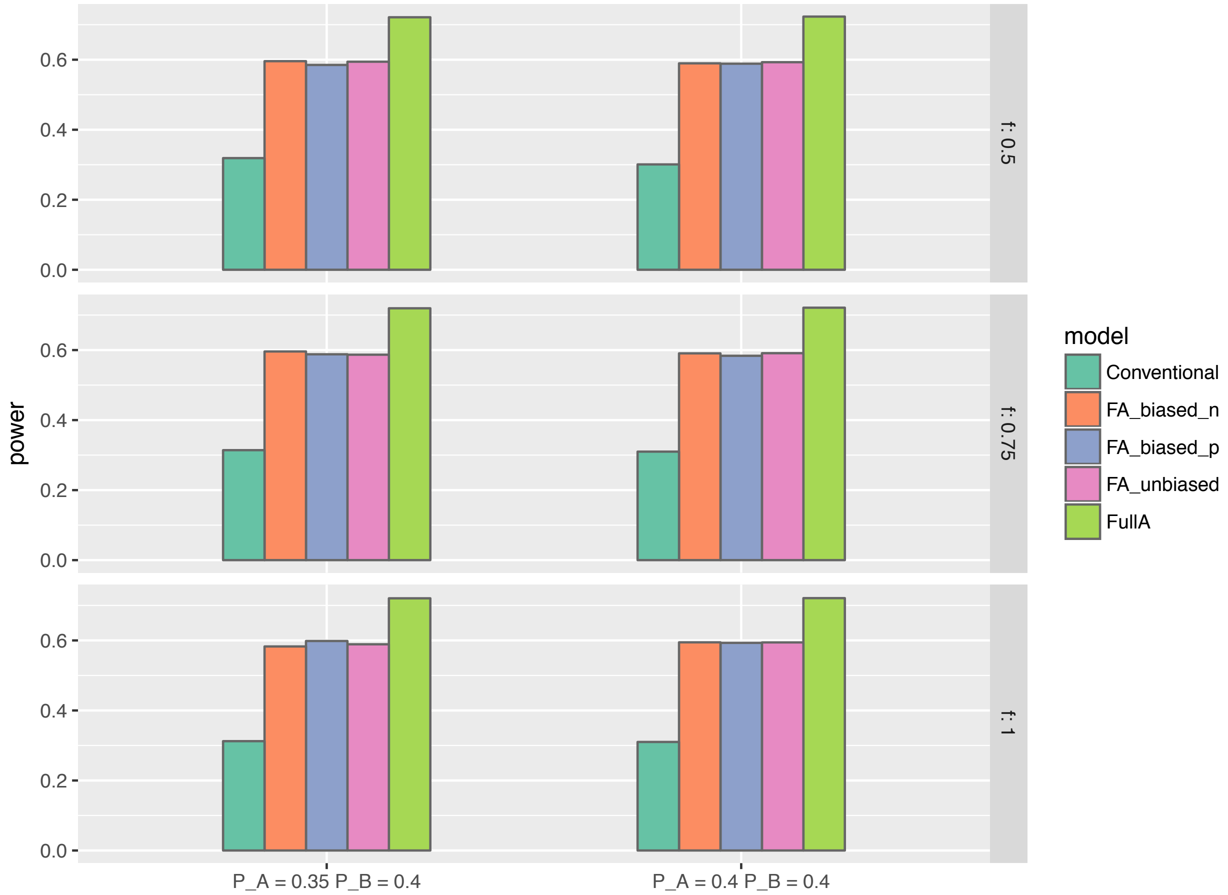

FA (fractional additivity); P_A (probability of response with A); P_B (probability of response with B).

Figure S.2: Probability of A&B being superior (y-axis) in relation to the number of interim looks (x-axis) in the non-adaptive trial simulation. The sample size between interim looks is fixed and allocation ratio is 1:1:1.

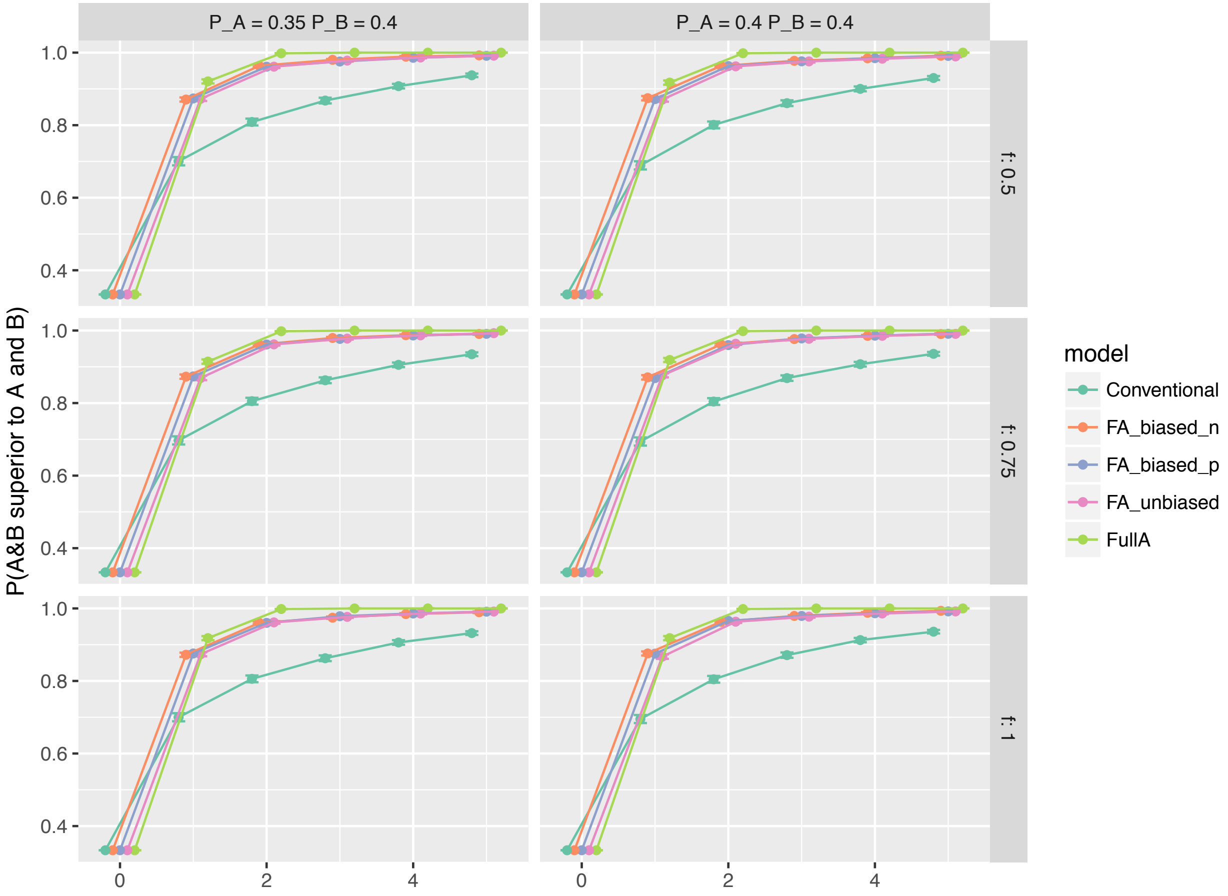

FA (fractional additivity); P_A (probability of response with A); P_B (probability of response with B).

Figure S.3: Probability of A being superior (y-axis) in relation to the number of interim looks (x-axis) in the non-adaptive trial simulation. The sample size between interim looks is fixed and allocation ratio is 1:1:1.

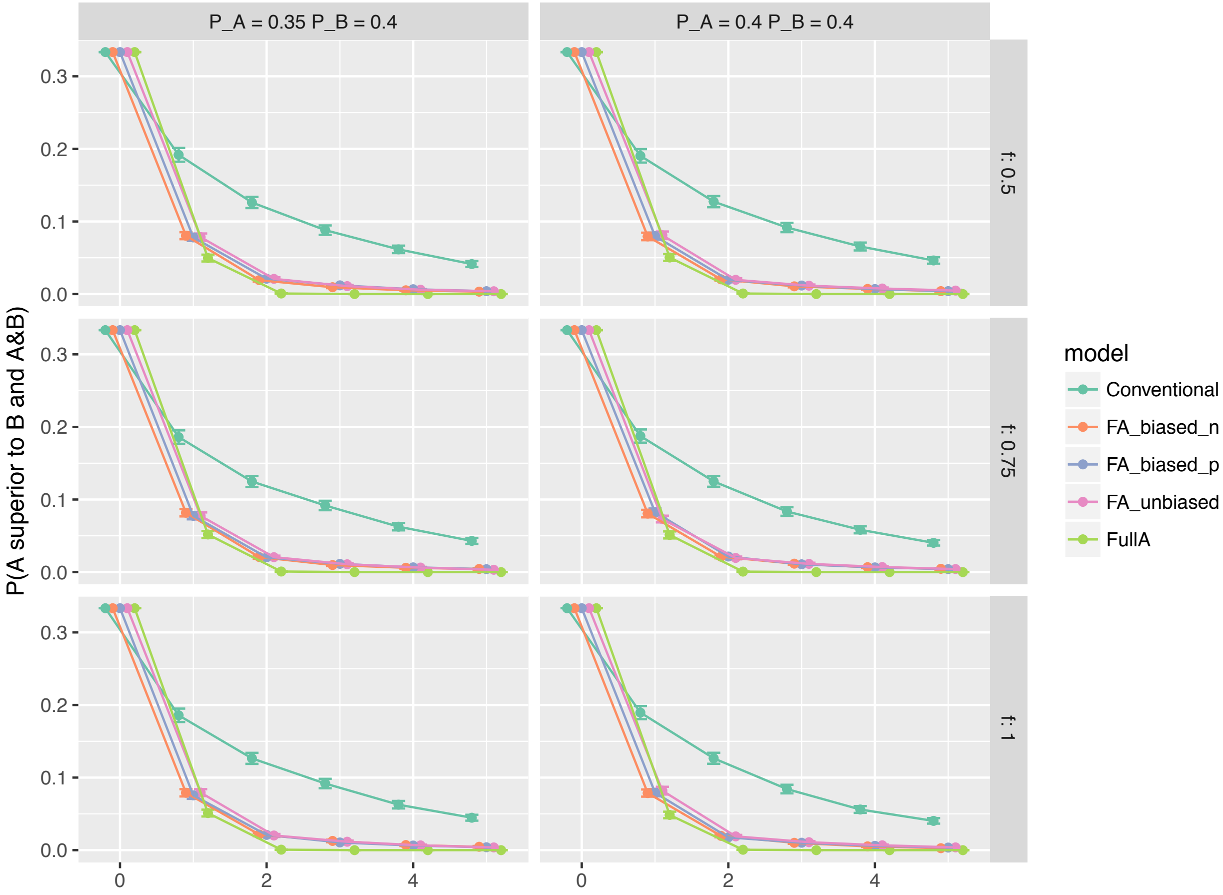

FA (fractional additivity); P_A (probability of response with A); P_B (probability of response with B).

Figure S.4: Probability of B being superior (y-axis) in relation to the number of interim looks (x-axis) in the non-adaptive trial simulation. The sample size between interim looks is fixed and allocation ratio is 1:1:1.

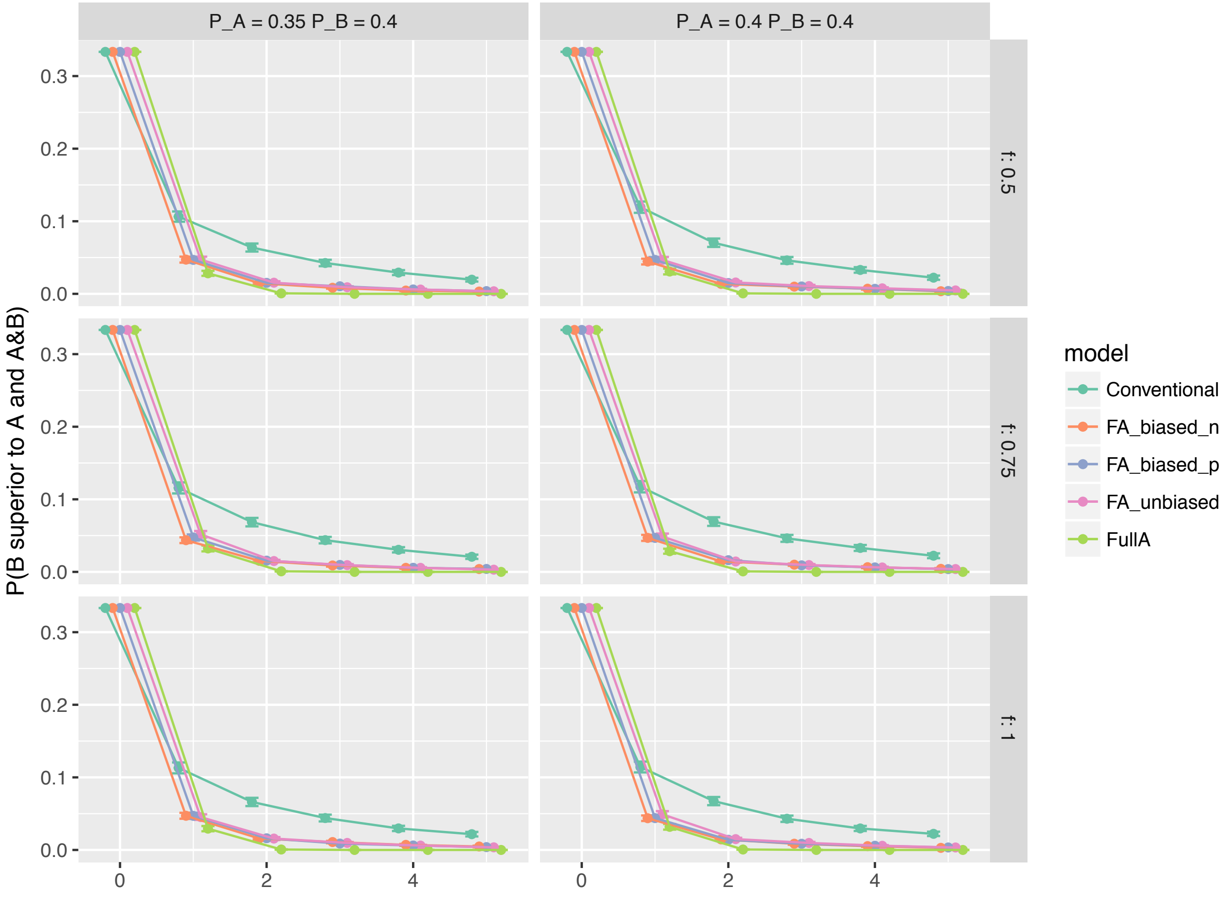

FA (fractional additivity); P_A (probability of response with A); P_B (probability of response with B).

Figure S.5: Evolution of probability of superiority (for all three arms) and posterior density estimates (for individual therapies A and B) for two example trials: an example where the data shows the expected fractional additive effect early (first row); one example in which fractional additivity is less pronounced due to random error and a larger number of batches is required to identify the superior therapy (second row).

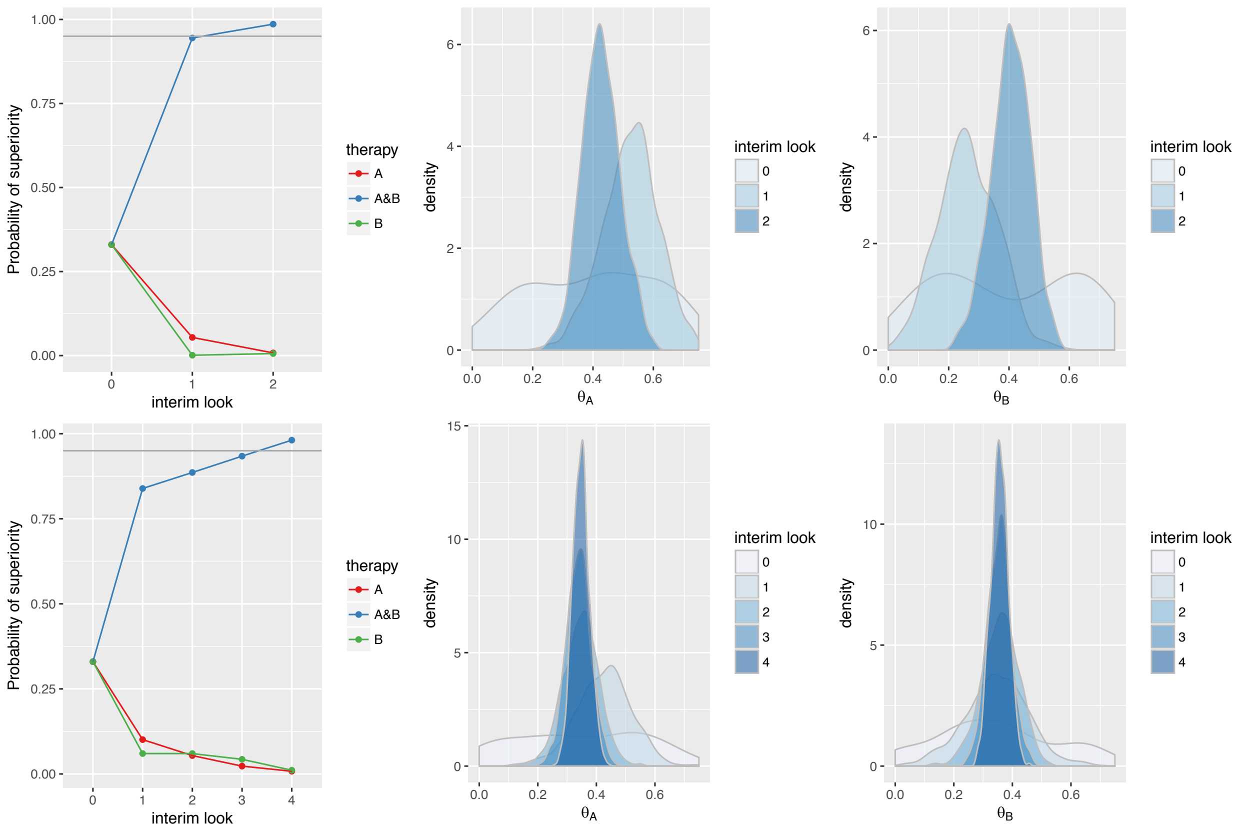

Figure S.6: Expected sample size in 4 simulation scenarios representing no or weak fractional additivity with biased priors on f with prior means 0.5 and 0.75, the conventional model (i.e, where $\theta_{A\&B}$ is treated as completely independent of θ_A and θ_B and all priors are non-informative) and a model with full additivity assumption ($f = 1$). The upper, middle and lower plots present the scenarios where the *true* fractional additivity parameter value (*f)* is set to 0, 0.25, and 1, respectively. The left plots present the scenario where the failure probabilities of treatments A and B are set to P_A=0.35 and P_B=0.40, whereas the plots to the right present the scenarios where P_A=0.40 and P_B=0.40. Note that the results for the conventional model and the full additivity model are averaged over a set of simulations with sample sizes specified by f equal to either of prior means 0.5 and 0.75.

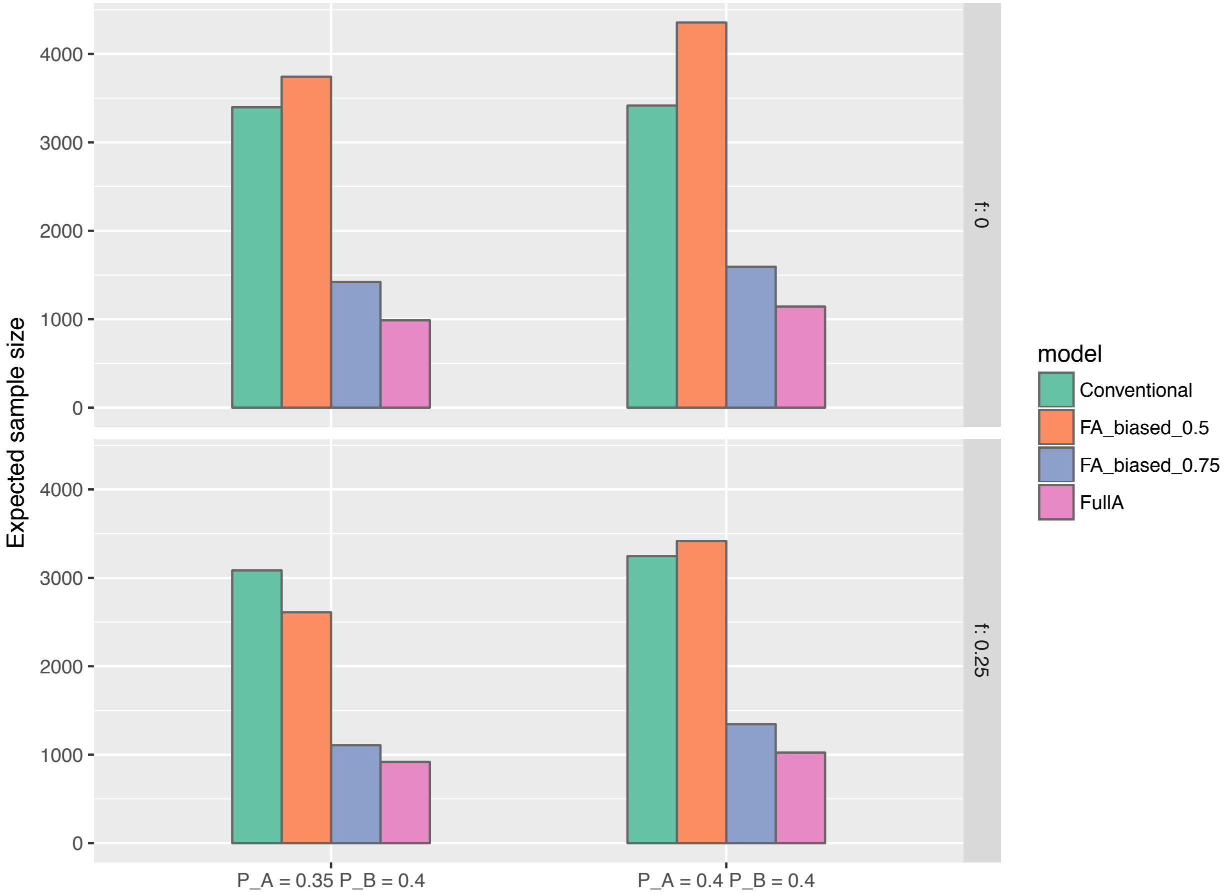

RMSE (root mean square error); FA (fractional additivity); P_A (probability of response with A); P_B (probability of response with B).

Figure S.7: Empirical cumulative distribution function for the probability of trial termination (y-axis) by cumulative Information Fraction (IF) of the required sample size. Note, each interim look accounts for 20% information fraction (e.g., the information fraction of 60% corresponds to the third interim look).

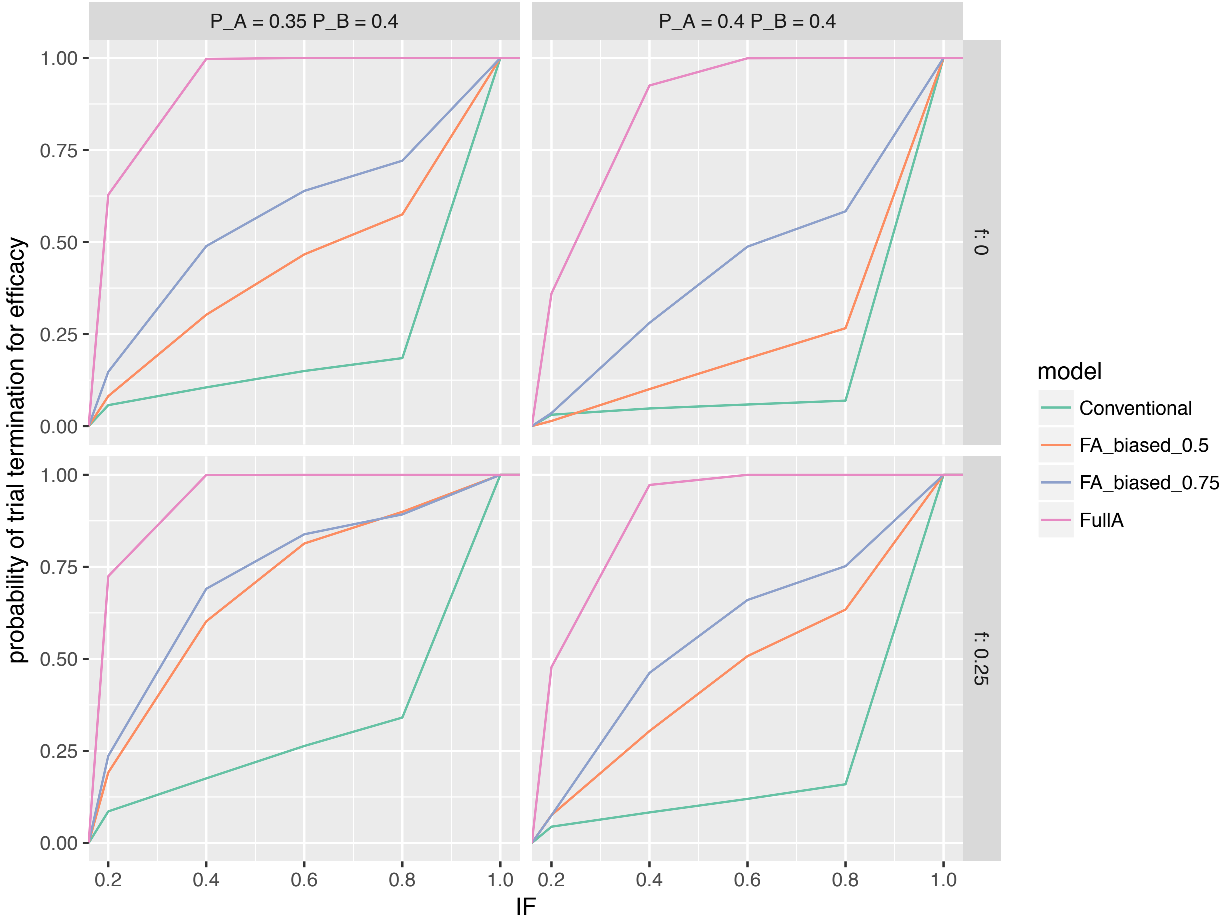

IF (information fraction); FA (fractional additivity); P_A (probability of response with A); P_B (probability of response with B).

Figure S.8: Root mean square error for the effect size of A&B calculated for the six different simulation scenarios for the proposed model with a biased and unbiased prior, the conventional model (i.e., where $\theta_{A\&B}$ is treated as completely independent of θ_A and θ_B and all priors are non-informative) and a model that assumes full additivity (*f = 1*). The upper, middle and lower plots present the scenarios where the *true* fractional additivity parameter value (*f)* is set to 0.5, 0.75, and 1, respectively. The left plots present the scenario where the failure probabilities of treatments A and B are set to P_A=0.35 and P_B=0.40, whereas the plots to the right present the scenarios where P_A=0.40 and P_B=0.40.

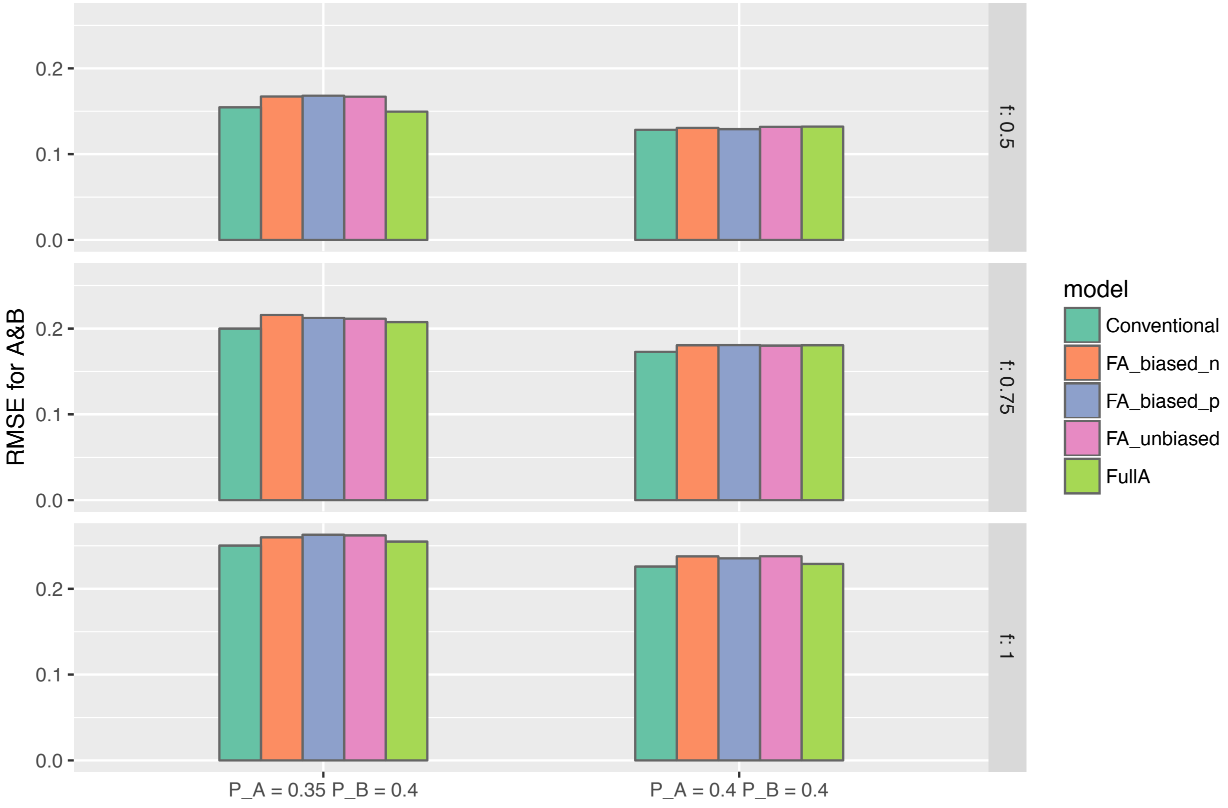

RMSE (root mean square error); FA (fractional additivity); P_A (probability of response with A); P_B (probability of response with B).

Figure S.9: Root mean square error for the effect size of A calculated for the six different simulation scenarios for the proposed model with a biased and unbiased prior, the conventional model (i.e., where $\theta_{A\&B}$ is treated as completely independent of θ_B and θ_B and all priors are non-informative) and a model that assumes full additivity (*f = 1*). The upper, middle and lower plots present the scenarios where the *true* fractional additivity parameter value (*f)* is set to 0.5, 0.75, and 1, respectively. The left plots present the scenario where the failure probabilities of treatments A and B are set to P_A=0.35 and P_B=0.40, whereas the plots to the right present the scenarios where P_A=0.40 and P_B=0.40.

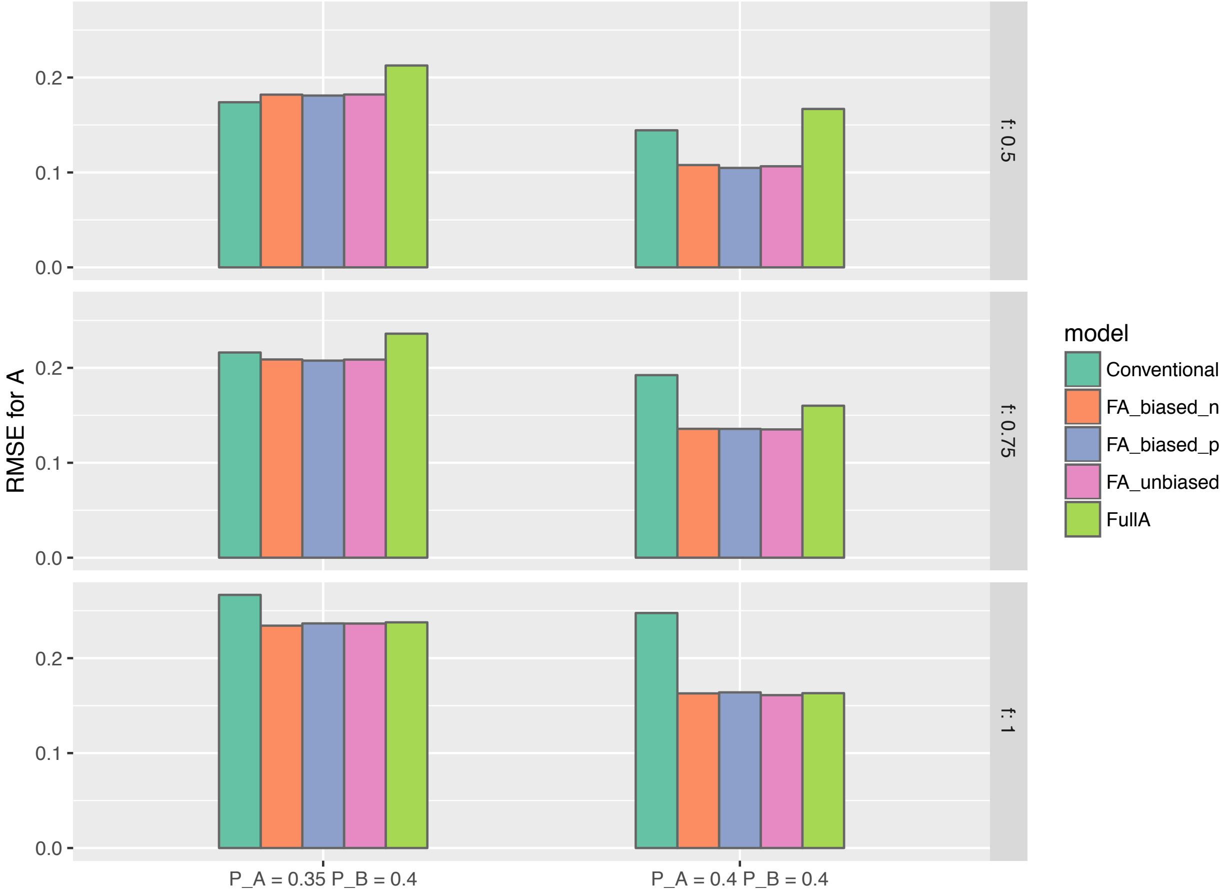

RMSE (root mean square error); FA (fractional additivity); P_A (probability of response with A); P_B (probability of response with B).

Figure S.10: Root mean square error for the effect size of B calculated for the six different simulation scenarios for the proposed model with a biased and unbiased prior, the conventional model (i.e., where $\theta_{A\&B}$ is treated as completely independent of θ_B and θ_B and all priors are non-informative) and a model that assumes full additivity (*f = 1*). The upper, middle and lower plots present the scenarios where the *true* fractional additivity parameter value (*f)* is set to 0.5, 0.75, and 1, respectively. The left plots present the scenario where the failure probabilities of treatments A and B are set to P_A=0.35 and P_B=0.40, whereas the plots to the right present the scenarios where P_A=0.40 and P_B=0.40.

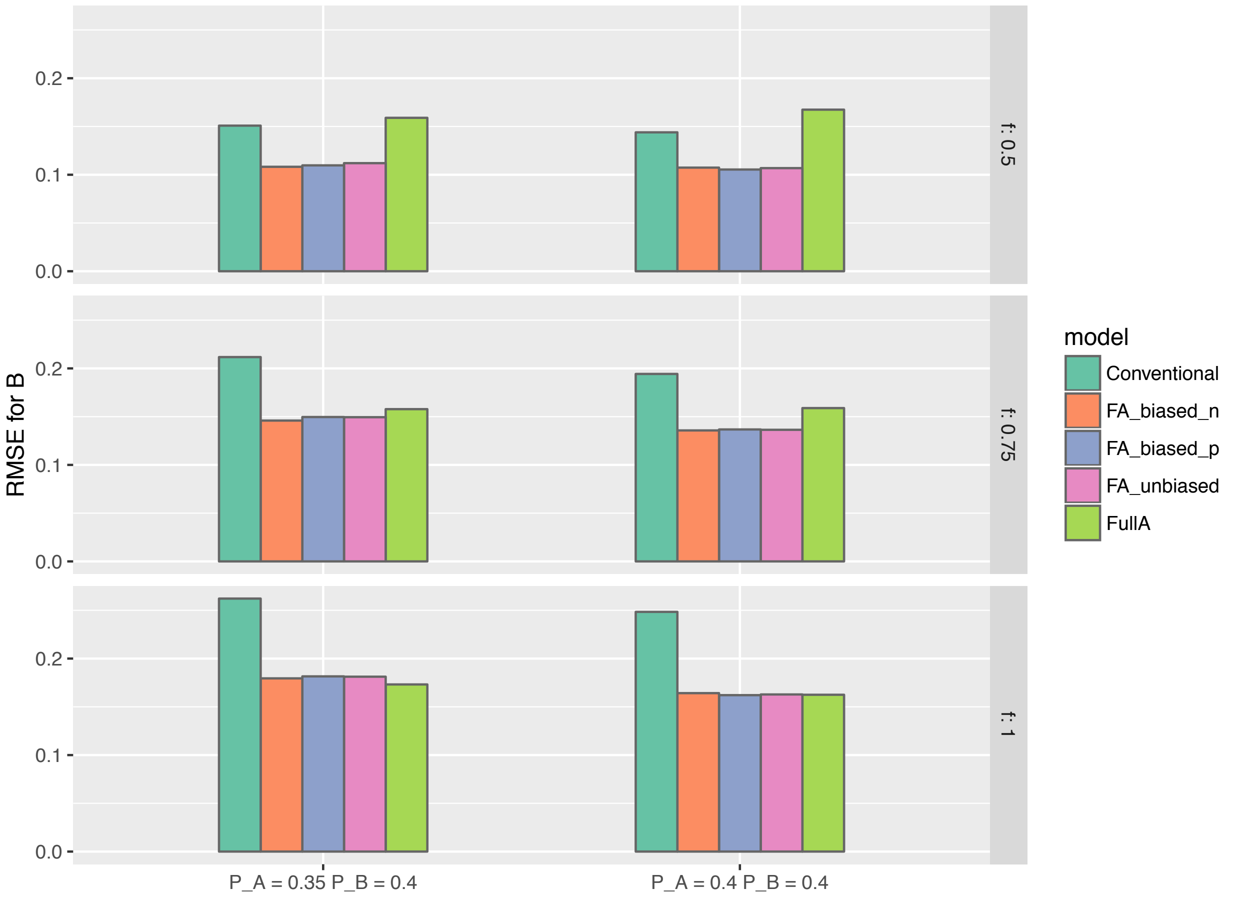

RMSE (root mean square error); FA (fractional additivity); P_A (probability of response with A); P_B (probability of response with B).

Figure S.11: Root mean square error for the effect size of A&B calculated 4 simulation scenarios representing no or weak fractional additivity with biased priors on f with prior means 0.5 and 0.75, the conventional model (i.e, where $\theta_{A\&B}$ is treated as completely independent of θ_A and θ_B and all priors are non-informative) and a model with full additivity assumption ($f = 1$). The upper, middle and lower plots present the scenarios where the *true* fractional additivity parameter value (*f)* is set to 0, 0.25, and 1, respectively. The left plots present the scenario where the failure probabilities of treatments A and B are set to P_A=0.35 and P_B=0.40, whereas the plots to the right present the scenarios where P_A=0.40 and P_B=0.40.

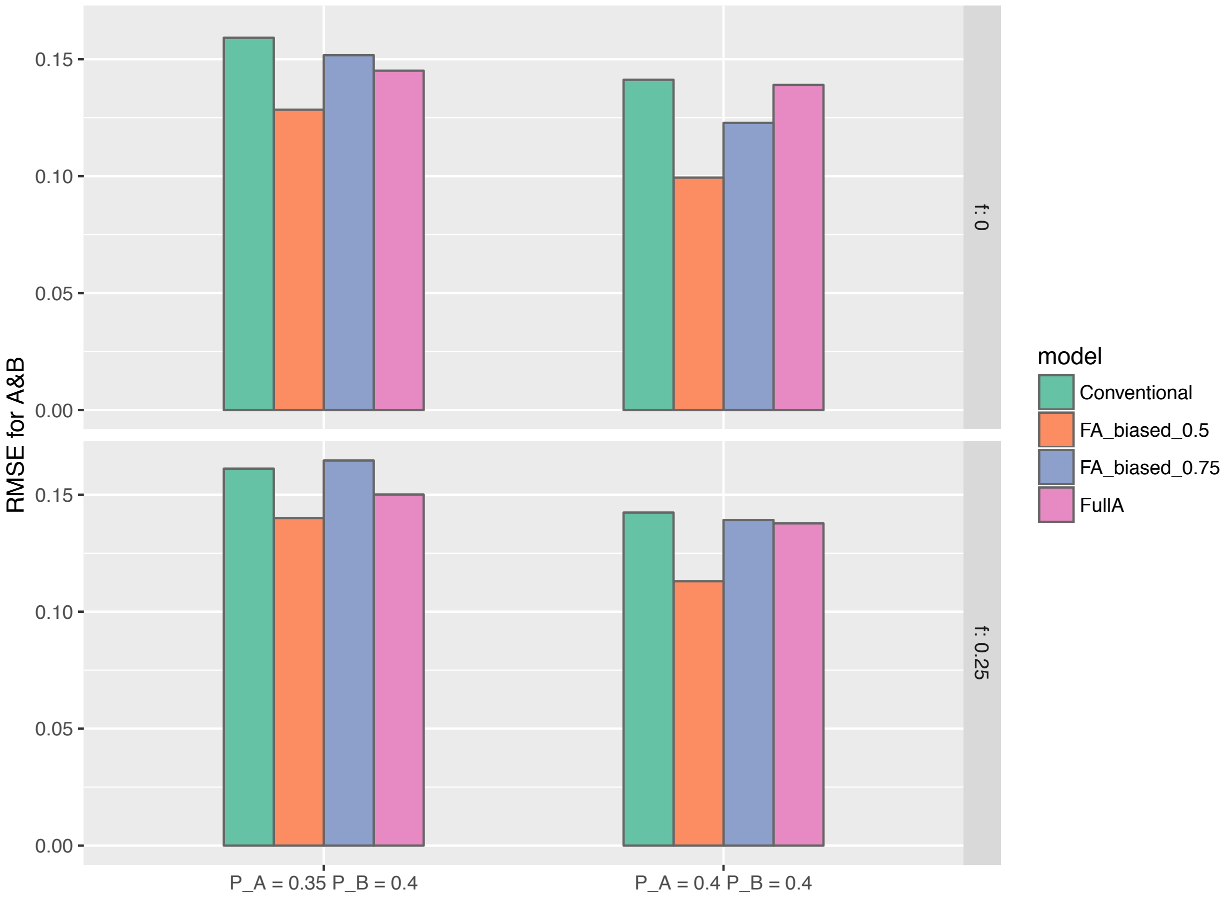

RMSE (root mean square error); FA (fractional additivity); P_A (probability of response with A); P_B (probability of response with B).

Figure S.12: Root mean square error for the effect size of A calculated 4 simulation scenarios representing no or weak fractional additivity with biased priors on f with prior means 0.5 and 0.75, the conventional model (i.e, where $\theta_{A\&B}$ is treated as completely independent of θ_A and θ_B and all priors are non-informative) and a model with full additivity assumption ($f = 1$). The upper, middle and lower plots present the scenarios where the *true* fractional additivity parameter value (*f)* is set to 0, 0.25, and 1, respectively. The left plots present the scenario where the failure probabilities of treatments A and B are set to P_A=0.35 and P_B=0.40, whereas the plots to the right present the scenarios where P_A=0.40 and P_B=0.40.

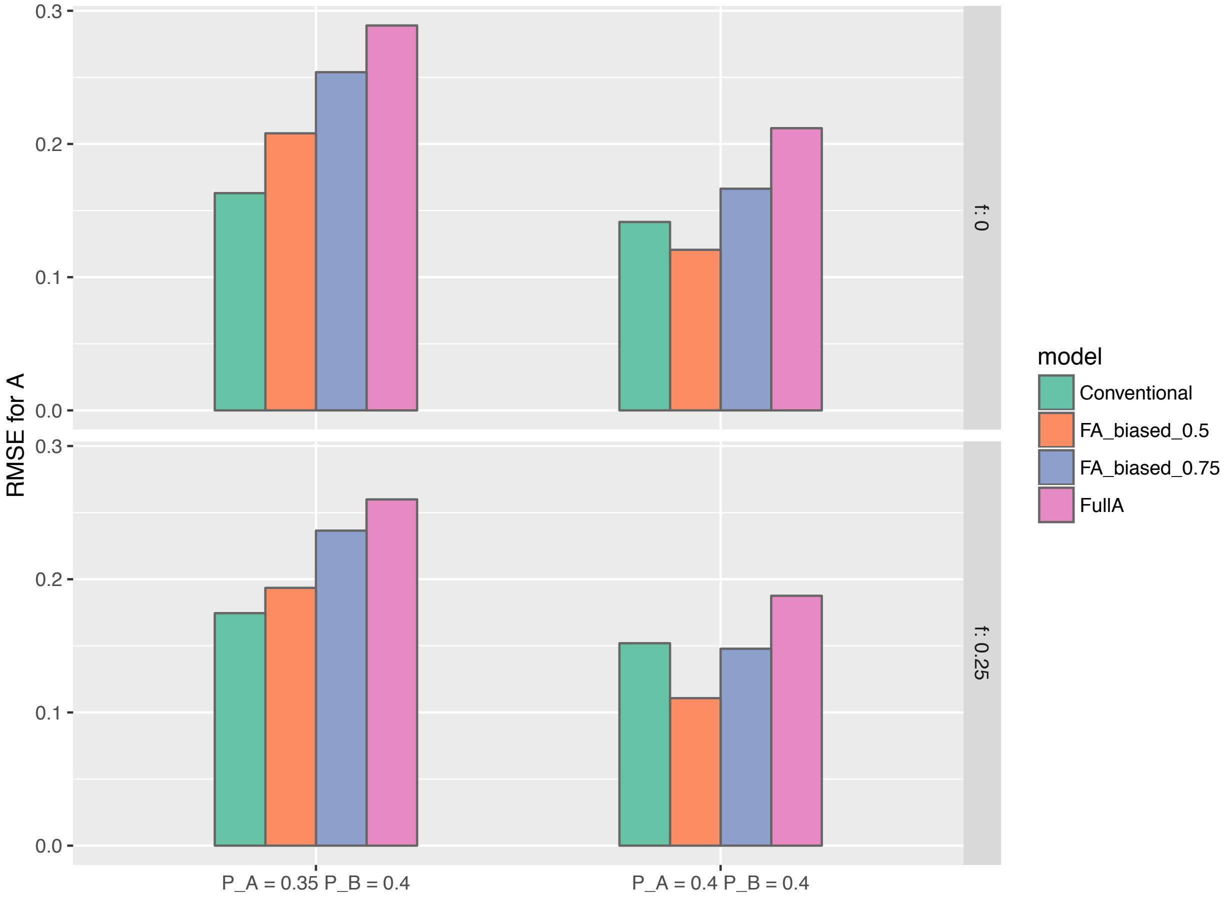

RMSE (root mean square error); FA (fractional additivity); P_A (probability of response with A); P_B (probability of response with B).

Figure S.13: Root mean square error for the effect size of B calculated 4 simulation scenarios representing no or weak fractional additivity with biased priors on f with prior means 0.5 and 0.75, the conventional model (i.e, where $\theta_{A\&B}$ is treated as completely independent of θ_A and θ_B and all priors are non-informative) and a model with full additivity assumption ($f = 1$). The upper, middle and lower plots present the scenarios where the *true* fractional additivity parameter value (*f)* is set to 0, 0.25, and 1, respectively. The left plots present the scenario where the failure probabilities of treatments A and B are set to P_A=0.35 and P_B=0.40, whereas the plots to the right present the scenarios where P_A=0.40 and P_B=0.40.

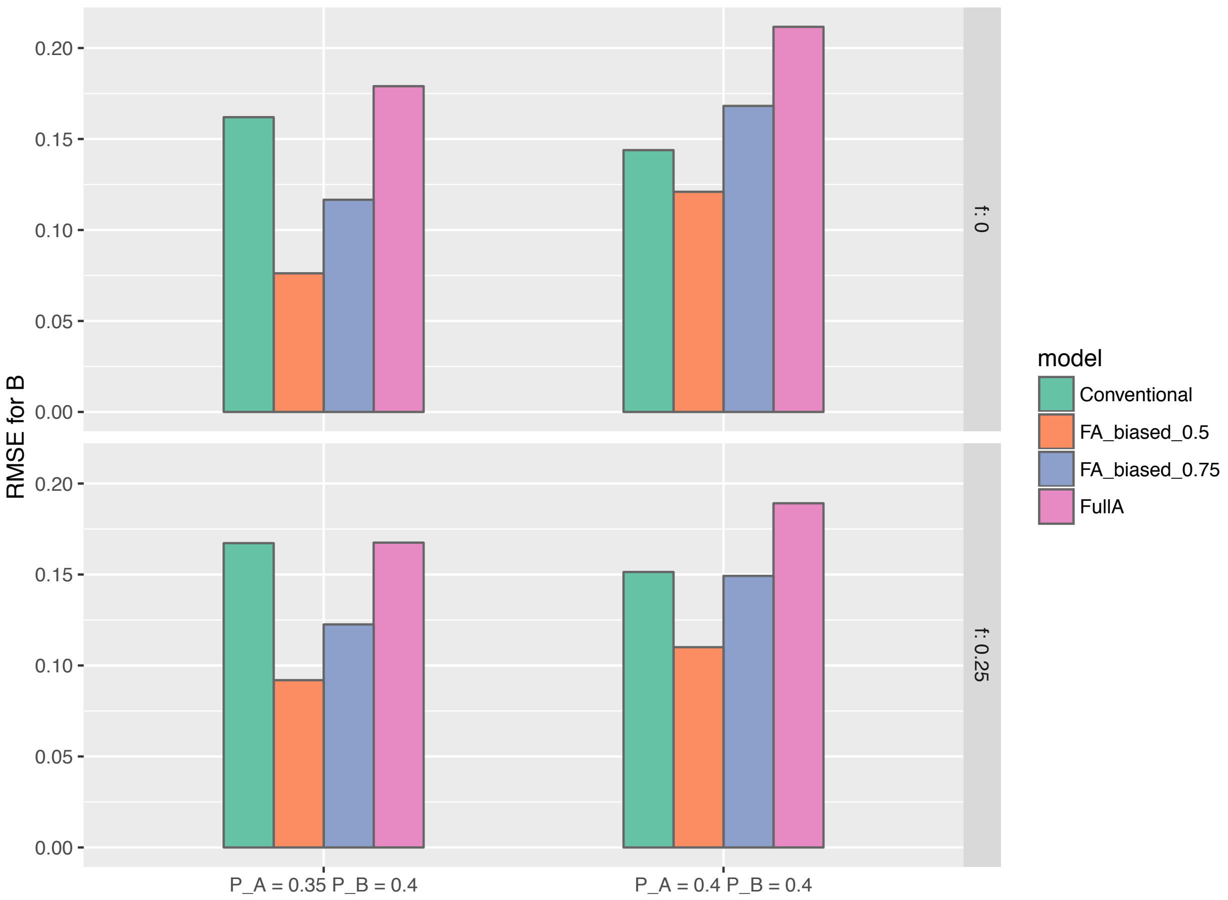

RMSE (root mean square error); FA (fractional additivity); P_A (probability of response with A); P_B (probability of response with B).

S.14 Stan implementation of the fractional additivity model:

```
data{
     int N;                                          // number of patients
     vector[N] treat_a;                              // binary vector: element n is 1 if patient n is treated by A
     vector[N] treat_b;                              // binary vector: element n is 1 if patient n is treated by B
     vector[N] treat_ab;                             // binary vector: element n is 1 if patient n is treated by A&B
     real theta_mean[2];                             // mean effect sizes for A and B
     real<lower=0> theta_sd[2];                      // sd of effect sizes for A and B
     real a0;                                        // prior mean for f
     real b0;                                        // prior sd for f
     int<lower=0,upper=1> y[N];                      // vector of responses: 0 if negative, 1 if positive
}
parameters {
     real theta[2];                                  // effect sizes for A and B
     real f;                                         // fractional additivity parameter
}
model {
     real mu_y;                                                          // latent continuous response
     real theta_ab;                                                      // effect size for A&B
     theta_ab = max(theta) + p * min(theta);
     f ~ normal(a0, b0);                                                 // prior on f
     for (j in 1:2) theta ~ normal(theta_mean[j], theta_sd[j]);          // prior on effect sizes of A and B
          for (n in 1:N) {
               mu_y = treat_a[n] * theta[1] + treat_b[n] * theta[2]
                           + treat_ab[n] * theta_ab;
                y[n] ~ bernoulli_logit(mu_y);                            // likelihood
  }
}
```
